# No man is an island: management of the emergency response to the SARS-CoV-2 (COVID-19) outbreak in a large public decentralised service delivery organisation

**DOI:** 10.1186/s12913-022-07716-w

**Published:** 2022-03-21

**Authors:** Mikael Ohrling, Karin Solberg Carlsson, Mats Brommels

**Affiliations:** 1 Department of Learning, Informatics, Management and Ethics, Medical Management Centre, Karolinska Institutet, Stockholm, Sweden; 2 Stockholm Healthcare Services, Region Stockholm, Box 45436, 104 31 Stockholm, Sweden

**Keywords:** Emergency management, Covid-19, Decentralisation, Dynamic capabilities

## Abstract

**Background:**

We wanted to better understand whether and how agility can be achieved in a decentralised service delivery organisation in Sweden. The pandemic outbreak of SARS-Cov-2 (Covid-19) provided an opportunity to assess decentralisation as a strategy to improve the responsiveness of healthcare and at the same time handle an unpredictable and unexpected event.

**Methods:**

Data from in-depth interviews with a crisis management team (*n* = 23) and free text answers in a weekly survey to subordinated clinical directors, i.e. unit managers, (*n* = 108) were scrutinised in a directed content analysis. Dynamic capabilities as a prerequisite for dynamic effectiveness, understood as reaching strategic and operative effectiveness simultaneously, were explored by using three frameworks for dynamic effectiveness, dynamic capabilities and delegated authority in a decentralised organisation.

**Results:**

Unpredictable events, such as the pandemic Covid-19 outbreak, demand a high grade of ability to be flexible. We find that a high degree of operational effectiveness, which is imperative in an emergency situation, also is a driver of seeking new strategic positions to even better meet new demands. The characteristics of the dynamic capabilities evolving from this process are described and discussed in relation to decentralisation, defined by decision space, organisational and individual capacity as well as accountability. We present arguments supporting that a decentralised management model can facilitate the agility required in an emergency.

**Conclusions:**

This study is, to our knowledge, the first of its kind where a decentralised management model in a service delivery organisation in healthcare is studied in relation to crisis management. Although stemming from one organisation, our findings indicating the value of decentralisation in situations of crisis are corroborated by theory, suggesting that they could be relevant in other organisational settings also.

**Supplementary Information:**

The online version contains supplementary material available at 10.1186/s12913-022-07716-w.

## Introduction

Management decentralisation of service providing organisations is based on the following principles: by delegating decision-making authority to frontline managers, local conditions and customer needs are better paid attention to and allowed to guide decisions, thus leading to greater responsiveness, better resource allocation according to needs, and consequently higher effectiveness and efficiency. There is a general consensus that these principles are shown to be valid on a systems level in public healthcare [1–3]. Our studies of one large healthcare service delivery organisation in Sweden indicate that the same mechanisms work intra-organisationally [4, 5]. When applying management decentralisation at the organisational level the main objective and focus are, consequently, on promoting organisational performance.

During the last twenty years this focus on performance and efficiency has led to wide-spread applications of service process improvement and the use of lean management approaches. The experience of lean in healthcare is ambiguous [6, 7]. One challenge is the complexity of healthcare organisations, which requires that management models, including lean, need to adjust to that complexity [6, 8].

Complexity in healthcare is increasing due to demographical and technological changes [9]. Complexity introduces new elements of unpredictability, which is a challenge to management. Rapid changes in the environment and surprising events will require an ability of organisations to swiftly adjust. A too narrow-minded focus on efficiency, performance and a lean service production might constrain such an ability and impede flexible responses. Consequently, the management literature emphasises that, in times of uncertainty, organisations need to be both lean and agile [10].

The Covid-19 pandemic was felt as an unpredictable event (although the threat of pandemics is always present). It required a very rapid adjustment of healthcare organisations to deal with the public health crisis that emerged. In addition, there was a need to mitigate the negative psychological impact reported on other patient groups, caused by the crisis [11, 12]. Consequently, the pandemic created conditions to study the role of management in relation to the capability of healthcare service organisations to flexibly and rapidly change their operations, i.e. to show agility in their responses.

The literature on rapid responses, flexibility or agility among decentralised healthcare service providers is limited. The Covid-19 pandemic provides an opportunity to study that issue. We were able to do so as a result of access to such a provider that applies management decentralisation [13]. We see as an advantage that we have performed earlier in-depth studies of the management model [4, 5]. To our mind, two alternative assumptions are both plausible: rapid adjustment to a radically changed environment can either be promoted by centralised, command-and-control management, or by a decentralised organisation with decision-making authority delegated to front-line managers, who flexibly and more rapidly will carry out the changes in operations needed. We further anticipate that the former will result in a static structure, whereas the second increases the likelihood that the organisation has the capacity to further adjust to new challenges and handle different demands in different parts of the organisation. We see the opportunity to scrutinise the second assumption in our case study.

### Study aim

The aim of the study is to better understand how agility can be achieved in a decentralised service delivery organisation. We formulated the following research question: What features of a decentralised healthcare service providing organisation might prepare the organisation to increase its responsiveness and handle an unpredictable and unexpected event such as the pandemic outbreak?

#### Theoretical framework

Our study was guided by three theoretical frameworks.

#### Dynamic effectiveness

**Fig. 1:**
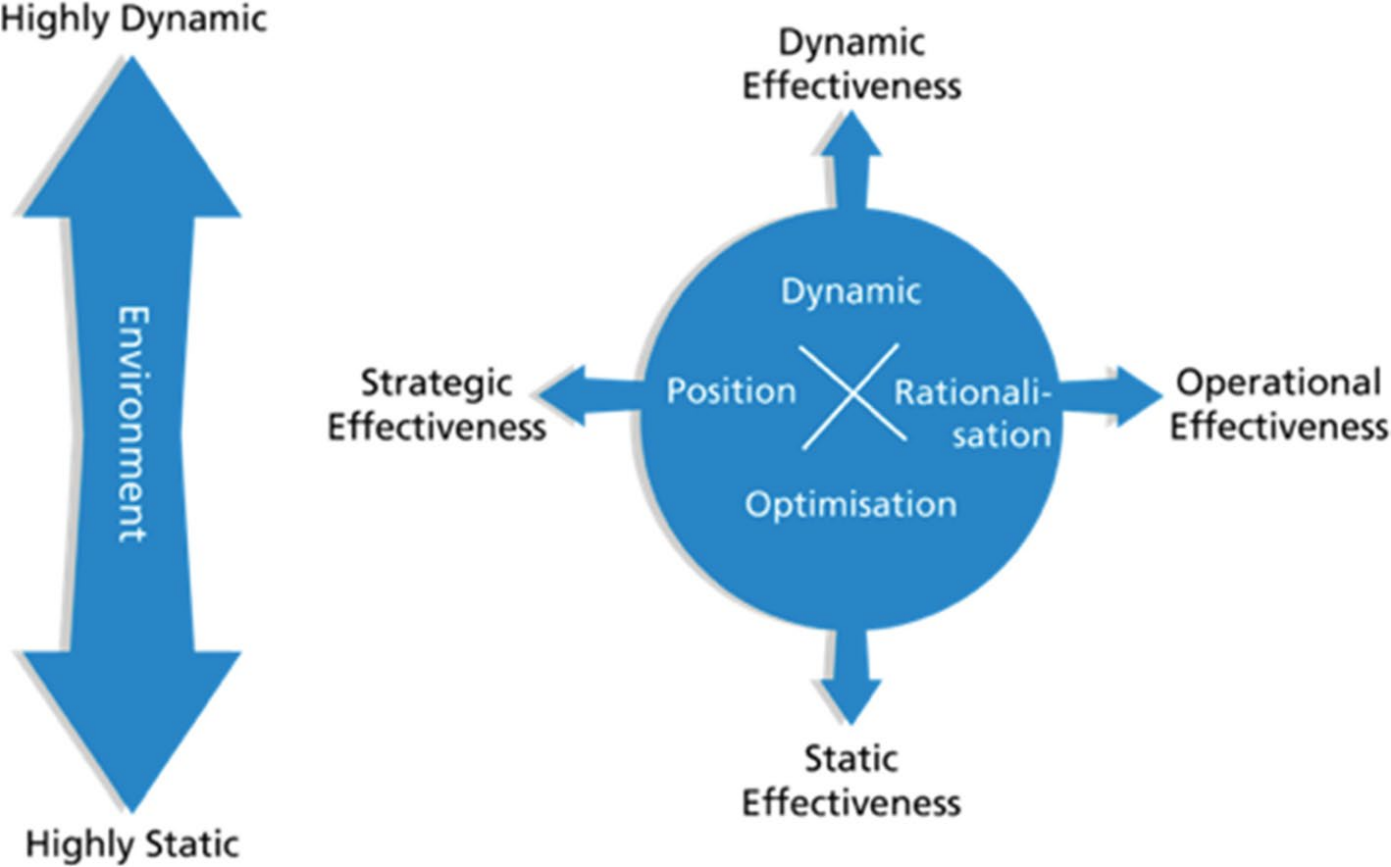
A conceptual model of dynamic effectiveness (adapted from Abrahamsson and Brege [14], p 103)

Abrahamsson and Brege [14] define dynamic effectiveness as “*how fast and well a company can go from one strategic positioning and productivity frontier to another*” (p 84). Such a capacity is important when the environment is unpredictable and rapidly changing, i.e., “highly dynamic”, by using the authors’ term. Dynamic effectiveness stems from an interplay between operational and strategic effectiveness. The former expresses that resources are utilised efficiently internally, and the latter that the organisation has the ability to adjust to environmental changes and seek new strategic positions. In addition, Abrahamsson and Brege [14] claim that high operational effectiveness is a precondition for strategic effectiveness. This conceptual model, exhibited in Fig. 1, advises us to explore whether the organisation in our case is perceived as dynamically effective, and to analyse what forms of operational and strategic effectiveness the organisation shows as potential explanations to such a capacity to adjust to an unpredictable environment.

#### Dynamic capabilities

Teece defines ([15], p 516) dynamic capability as *“the ability to integrate, build, and reconfigure internal and external competences to address changing environments.”* This is an efficiency-based framework in the field of strategic management. The focus is on competitive strategy and the framework illustrates essential elements to be explored and exploited to enable new positioning [16]. The framework identifies three capabilities, following one another consecutively, that promote that ability to address a changing environment. “Sensing” is the ability understand the situation, “seizing” the readiness to utilise new opportunities offered by the changes, and “managing” the internal processes of readjustments to grab those opportunities [15]. The capabilities constitute the micro-foundations, which are defined as concrete activities within the three capacities that make the organisation dynamic [15]. We will explore if the case organisation shows such capabilities possible to relate to the degree of the dynamic effectiveness observed.

#### Decentralisation

We conceptualise management decentralisation in a framework that we have described in a previously published scoping review, as developed from Bossert’s [1, 3] original decision space model, in order to fit service delivery organisations. It illustrates the interaction between delegated management authority (“decision space”), accountability, and individual and institutional capacities in the organisation, and their impact on organisational performance [1, 3, 13]. This model will be utilised to assess if there is a congruence between dynamic capabilities found in the case organisation and its decentralised management model.

## Methods

### Study design

In order to capture in depth the subjective points of view of persons involved in emergency management in the case organisation on the organisation’s response to the Covid-19 pandemic in the winter and spring of 2020 [11] we chose a qualitative research method to study our case. We performed semi-structured interviews [17] with all members of the emergency management team. In addition, we analysed free text answers from a weekly survey sent out to unit managers responsible for adjusting their operations to achieve proper responses to the pandemic.

### Study setting

We studied the Stockholm County Health Care Services delivery organisation in Stockholm, Sweden (Swedish abbreviation SLSO) [18]. It is a tax-financed public organisation that provides healthcare in mental health services, primary care, advanced home healthcare, as well as several specialist clinics outside hospitals. Sweden has 21 regional self-governing authorities that have the primary responsibility for healthcare provision. They are funded by regional taxes and run by regional councils, elected by popular vote. The region integrates contracted private providers and region-owned service providers into a comprehensive system [19].

SLSO is financed and controlled by the largest region in Sweden, the Stockholm Region, and provides primary and community care for 2 million inhabitants with a staff of 12,000 employees, a yearly turnover of 1,5 billion euros, 6 million outpatient visits and 500,000 in- patient days per year. Founded in 2004, SLSO became a conglomerate of all regional public primary and community care outside the hospitals in the region. It is one of the largest healthcare organisations in Sweden. Clinical directors of all service providing units (“profit units”) across specialities and geographies have, from the beginning, been given a significant amount of delegated management authority, being responsible for budgets, staff and the procurement of consumables and external services as well as being held accountable for their actions and coordinated in a line structure built on value- and trust-based governance. This decentralised model is called the “enterprise within the enterprise”. SLSO service provision is funded by service contracts with each unit. Relations with the regional Purchasing Office are handled directly by the clinical directors with the support of SLSO’s top management team [18]. These clinical directors will be called “unit managers” in the following.

The study is performed during the pandemic of SARS-CoV-2 (Covid-19) in the Stockholm region. The management of healthcare emergencies and catastrophic events is the responsibility of self-governing regions. The Swedish government issues binding legislation on the national level, and national health and disease control authorities make recommendations. The first wave started in March and began to level off in May 2020 [11]. The task of the SLSO emergency management team was the operative coordination of all healthcare outside hospitals in the region, no matter private or public, and to collaborate with hospitals and municipalities. This coordination covered 217 primary health care centres and 10 local emergency centres, nearly 1,000 geriatric beds at 10 hospitals, more than 1,000 beds in mental health services, 3,200 patients in advanced home care, 250 beds in palliative care and medical services to over 15,000 residents in 400 elderly care homes run by the 26 municipalities.

### Study participants

Three researchers individually interviewed all 23 persons (12 men and 11 women) involved in the SLSO emergency management team. They had specific functions, often based on their previous experience and expertise, as required by the Region’s crisis management model. The profile of the emergency management team members is as follows: all have higher academic education (medical in the case of line-managers, economic or social sciences for administrative managers and specialists trained in pharmacy, communication, IT and psychology). All have more than ten years of experience in their field. The functions assigned to emergency team members were: 1. Staff deployment and HRM, 2. Security and legal affairs, 3. Situational assessment and response management, 4. Material supply and logistics, 5. Analysis and planning, 6. Technical communication and management support, 7. Communication and mass media relations, 8. Expert support including financial management, 9. Collaboration with and coordination of external providers, 10. Crisis management support.

The emergency management team for SLSO met twice daily from the 1 March 2020 to perform the ten specified functions stated above [20, 21]. The main objectives of the emergency team and the functions were to establish and operate the emergency management structure and systems, and run the operations to mitigate and stop the spread of the virus [11].

All but three interviews were conducted via video due to the ongoing pandemic. They were recruited by either telephone contact or e-mail. All chose to participate. The interviews took place in May and June 2020.

All 108 unit managers in the SLSO services, were sent a weekly survey by email from March to June 2020. All managers have a clinical background in medical specialties. The weekly participation rate varied from 22% (last week) to 63% (first week), with a mean of 52% and a median of 56%.

### Data collection

In total, 23 individual semi-structured interviews with the emergency management team members were performed by three experienced researchers. Most interviews lasted around one hour each (ranging from 20 to 70 min). The interview guide addressed the participants’ experience of working in the emergency management team, factors that facilitated or obstructed the work, lessons to be learned for a future crisis, and insights for the organisation and healthcare system as a whole. The interview guide is presented as Supplementary 1.

Each interview followed a standard procedure. We informed the participants about the purpose of the study, that participation was voluntary, that they could withdraw from the study at any time, that all data would be handled confidentially, and that maximum effort would be made to maintain anonymity when presenting the data. Each participant gave their oral informed consent for recording the interview twice: once before the interview started and once after the recording started. We recorded and transcribed each interview *verbatim*.

Thirteen weekly surveys were sent out to unit managers during the period from 28 March to 30 June 2020. The survey covered questions regarding their urgent needs and experiences concerning the first wave of the Covid-19 outbreak. Both quantitative and qualitative data were collected as feed back to the emergency management team and to enable rapid actions.. Several survey questions had a free-text box enabling participants to give detailed answers.. The survey is exhibited in Supplementary 2.The unit managers were very busy and under pressure, which is why we chose to analyse their free text answers for our study, rather than ask them to include time-consuming interviews in their busy schedules.

### Data analysis

**Fig. 2:**
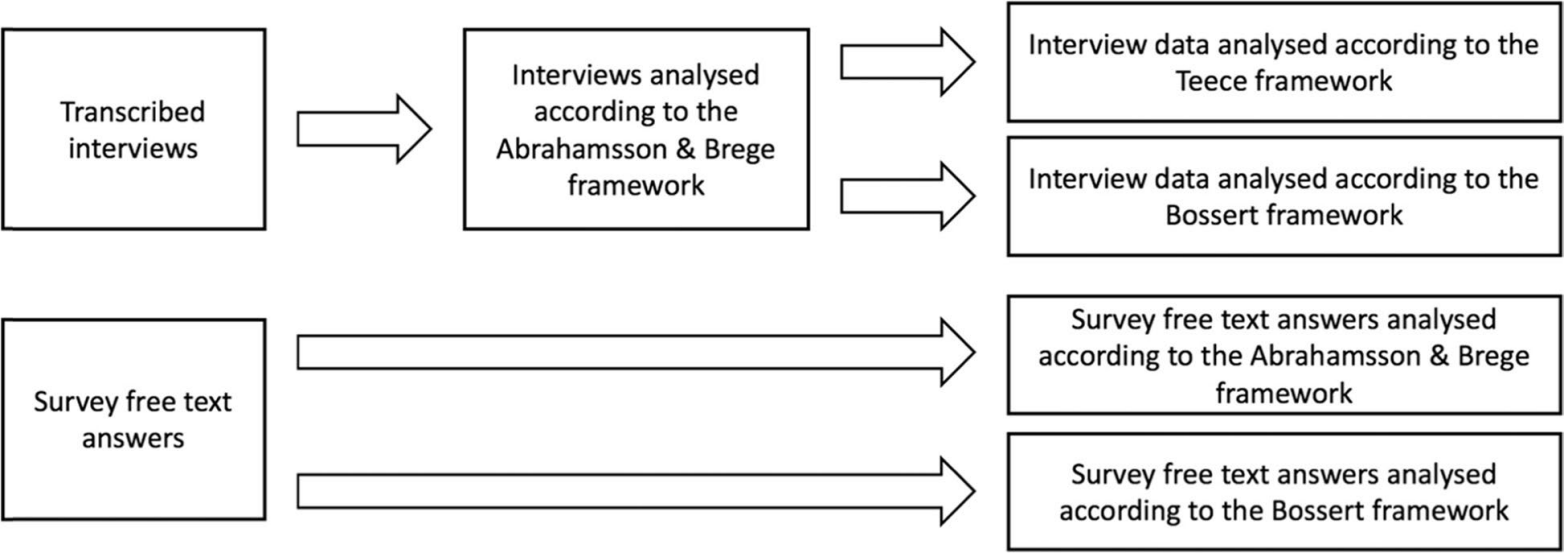
Overview of the data collection and analysis process

Each interview transcript was read to get on overview of the material before systematically analysing the data. (see Fig. 2). Based on the Abrahamsson and Brege framework [14], a codebook was created with pre-selected categories in NVivo 12 [22] (QSR international 1999) in order to analyse the material.Directed content analysis was used to categorise identified manifest and latent meaning units and also to code interesting material not related to the interview guide, derived inductively and collected under a separate category (“Other”) [23, 24]. Latent meaning units were identified and analysed during the interviews and in the transcripts by noting moods, such as laughter and tone of voice,. The audio recording was checked if there were doubts regarding in what tone something was said. If there were uncertainty about which code to use for the latent message, the larger context in which something was said, such as reading what was said before leading up to the latent meaning unit of interest, guided the selection. At a later step, the interview material was analysed in parallel using the Teece framework [15] and the revised Bossert decision space model [1, 3, 13].

Meaning units were identified in the free text answers to main and follow-up questions of the survey and analysed using the Abrahamsson and Brege conceptual framework [14]., Those were categorised as expressing either strategic or operational effectiveness. The meaning units were, in parallel, analysed according to the revised Bossert decision space model [1, 3, 13]. Meaning units that were ambiguous too “unit specific” were excluded, the latter as not being related to the organisation’s core work. The numbers and percentages of meaning units for each main question relevant for this study are available upon request.

To ease data processing and improve transparency, the coded meaning units were condensed, translated into English, and sorted in framework matrices in NVivo 12 [25]. The themes derived from the categorised findings are presented below and further described by quotations. Detailed information is available upon request. Interview data were checked with corresponding information found in protocols and plans of the crisis management team (data triangulation) [26].

### Ethical considerations

The interviewees were informed about the purpose of the study, that participation was voluntary, that they could withdraw from the study at any time, that all data would be handled confidentially, and anonymity was granted in all presentations of the data collected. Each participant gave their oral informed consent twice, before the recording and at the start of the recording as a documentation of approval.. Due to the pandemic the interviews were performed via video call. Each interview was recorded and transcribed verbatim. The unit managers were informed that the weekly survey would be used for research purposes and were granted that no individual answer could be identified. All methods were performed in accordance with the research ethics guidelines and regulations including the verbal consent to participate in the study.

This study has been performed as a part of a larger project on “Implementation of management and organisation response to the COVID-19 outbreak: a study of the crisis organisation in Stockholm County’s healthcare area”, the research plan of which has been evaluated and endorsed by the Swedish Ethical Review Authority on 8 April 2020 (Dnr 2020–01,521).

### Methodological considerations

Our material consists of two data sets. First, we performed extensive interviews with all members of the emergency management team. Secondly, we collected opinions from all unit managers expressed in free text answers in weekly surveys performed during the study period.

The models provided all authors with a structure to sort, code and analyse the findings of the interviews that were carried out by the second author and two other trained researchers. The analysis was made using anonymised and consolidated meaning units. By that procedure the personal integrity of all interviewees and the confidentiality of interview statements were protected. That enabled the first author, who is the chief executive of the organisation studied, to take the lead in the subsequent analysis.

## Findings

### Interviews with emergency team members

When first applying Abrahamsson and Brege’s conceptual model [14] to the interviews with the emergency management team 665 of the 671 meaning units formed were categorised as indicating a highly dynamic organisation and 6 as static.. In the second analysis, thirty-seven categories with sub-categories were further identified, fitting the three dynamic capabilities of the Teece framework [15], i.e. “sensing”, “seizing” and “managing”. A fourth dimension was inductively identified and labelled “perceived outcomes”. These categories were finally matched to the three dimensions of the revised Bossert model, “delegated authority”, “institutional and individual capacity” and “accountability” [1, 3, 13]. The findings are presented below and illustrated with quotations.

### Unexpected situation met by a highly dynamic organisation

The managers expressed that the nature of the pandemic and the perception of an acute emergency situation urged a rapid response. A positive attitude, right competence in the organisation, improved collaboration, trust and no blame-game in combination with a large decision space and less urgent and important issues being set aside were all factors that facilitated the redesign of the ordinary management group into an emergency management team. A clear target and the region’s recommended crisis management procedure to support the emergency management team were important facilitators of the rapid transition.

*“The crisis management model has worked excellent, as clear as it can be, you direct with full hand, you report back and you have direct contact upwards and the way back in the same order”. (21)*

*“It was good, that there was no prestige that the structures should be in a fixed way from start, now you could find the structures and procedures that functioned best over time, and it was clear early, which were the key functions in the emergency organisation.”(20)*

Complaints were made about ambiguous mandates on system level from the regional emergency organisation. The absence of directions complicated the situation, delayed decisions and led to waste of resources. This was mitigated with decisions taken by the SLSO emergency management team. Early in the emergency processes it became obvious to the SLSO team that a coordination mechanism for all regional providers in primary and community care, no matter public or private, had to be established. This was done by SLSO without formal approval in order not to delay a prompt response to an obvious need.

*“SLSO realised the need of a consolidated operative emergency management team operating outside the acute hospitals within the whole region, and since no one else took that role SLSO did it, and the impression was that no one was against it [at regional level]”. (7)*

The engagement and rapid problem solving, thanks to right teams and competence with clear roles and assignments, contributed to the coordination both on system and organisational levels, and the high level of dynamic effectiveness. The emergency management team had daily follow-ups to adjust efforts made, utilised its own internal competencies to support the functions of the larger health system, actively contributed to enhanced collaboration between several actors in their own organisation as well as throughout the health system, and rapidly implemented digital solutions to care delivery. The proactivity and local decision making were needed to create conditions that made it possible for the regional emergency organisation to function properly.

### Sensing is rapidly understanding the unexpected

#### Early resources were crucial for understanding

The managers described how early mobilisation of resources were crucial for an increased ability to analyse and understand the situation. The reason to have been assigned a certain role in the emergency management team was determined based on the expertise and experience of the person. Some were recruited from outside the ordinary management team as a means to reinforce the ability to understand details needed to know in operations and for networking outside the organisation. A clear target flexibly adjusted and clearly communicated to everyone wase important to create a common understanding. A positive attitude without unnecessary prestige of the team members and a lot of trust to one another enhanced cooperation and cohesion.

#### System established for understanding

The understanding of the situation required scanning, exploring, searching and learning by observing changes in the environment. Systematic collaboration was enhanced, internally by scheduled and frequent meetings and externally by utilising the organisation’s since long established networks in the region. However, initially the mandate of the emergency management team of SLSO was unclear in relation to private providers and the regional emergency organisation.

“It took some time before we understood the scope of our responsibility, where it ended and when it was handed over to someone else”. (20)

#### Information activities were reinforced

Information was crucial to understand the situation and what was needed to be done. Shared situation plans and clear goals for the activities facilitated rapid, correct and shared information in the emergency management team. Special communication channels on the web for rapid spread in the organisation were set up. Senior management shared their insights about regional emergency activities with their local colleagues. Capacity planning was a challenge due to lack of some real time data.

### Seizing is rapid response and actions

#### Reinforced emergency management team

The managers described how the management team was reorganised into an emergency management team, following the regional crisis plan which required a more coordinated, effective and focused teamwork with external experts where needed. The meetings were initially twice daily to ensure that information was communicated fast. Important changes to SLSO operations that normally would have needed a long time to make were now realised rapidly and meetings were readjusted after what had to be done in a flexible way. Crisis support was increased for patients, but also for staff. Special support for managers was available.

#### Increased collaboration with stakeholders

Initially, extensive adjustments of management procedures were intended to reinforce the emergency management team but also to coordinate activities and information exchange with other stakeholders. Voluntary and other non-healthcare organisations were mobilised and used to substitute healthcare staff. External experts and managers with crucial know-how were recruited to the emergency management team which increased the ability to make rapid decisions. Task shifting occurred in several services and competences were directed to areas where most needed in the system. For example, a special unit for infection tracing was set up. Initially the conditions to solve problems in the system were not in place. Managers also described how SLSO started early to coordinate actors within the regional system even before the formal mandate was issued by the Region after a request from SLSO.

#### Rapid and effective decision processes

Borders between service units lost importance. The shared focus and goals made it possible in a flexible way to establish new processes to mobilise needed capacity. The emergency situation in itself was a factor that motivated rapid adjustments. Focus on less important matters were set aside. Collaboration between units was smooth due to frequent meetings and decisions could be reconsidered whenever needed. Prompt reporting and feed-back for instant learning and readjustments turned out to be crucial. Problems could be solved more rapidly thanks to more appropriate organisational constellations, well defined roles and effective networking, both internally and at systems level. A constraint was lack of some real time data. Unclear or less well-informed decisions by the regional emergency organisation led to waste of resources.

#### Specific roles were identified

Each emergency management team member was assigned a task according to her or his special competence. Those tasks corresponded to the “functions” of the emergency organisation. (The functions are presented in the section on Study participants). The right competences could be mobilised fast from within the organisation which facilitated changes needed to the benefit of the whole regional system. Existing well-known networks could easily be accessed and utilised in crisis management activities. The members in the emergency team had to work fast and be pragmatic. The medical competence and experience of those senior managers and experts were considered crucial success factors. As an example, at the start there was a need to prioritise medical issues and rationing of personal protective equipment (PPE) and other material, which was managed by those with medical expertise. The emergency management team members’ trust in one another, the lack of panic, the team’s focus, decision capacity, experience, ability to listen and control ofthe situation were mentioned as success factors, too. Members of the emergency team expressed that they were confident and proud to contribute and felt a lot of trust and support from one another.

#### Digitalisation processes exploded

One important strategic decision was to rapidly scale-up digital consultations and the provision of e-health information to both the public and staff. The digitalisation process had met some resistance, but became over night the normal contact way, a change that in normal times would have required years to get in place. The digital tools also made it possible to work from home for those who could and with good results.

“The increase was 1 117% for digital consultations in primary care and 175% in psychiatry”. (19)

#### Information flow intensified

Information from different sources increased and was collated and displayed on the intranet to provide staff with a good overview of the situation. Special information channels were designed with the involvement of the whole emergency management team and spread rapidly and timely to private and public managers in primary and community care and to contact persons in the municipalities—long before the formal mandate was given to SLSO.

#### Extended responsibility was granted

The managers also expressed that there were rapid changes on the health systems level. Some primary healthcare centres changed into designated “infectious nodes”, assigned to receive patients with suspected Covid-19 in a safe process without risks to other patients. This model was suggested by SLSO to the Region which endorsed it. SLSO was provided with the formal mandate to coordinate all operations in private and public primary and community care as well as municipalities in the region. A geographic “cluster organisation” was established to coordinate the operations. When the number of inpatients increased dramatically, a field hospital was established and SLSO got the assignment to run that operation. Within two weeks SLSO had recruited, trained and prepared the hospital to receive patients. Those plans had never to be activated. Local links and flexibility made it possible to handle the shortages of PPE, as the coordination was given to SLSO and local stocks were built up.

*“During the first weeks SLSO worked with its own services, but this was gradually extended to include the coordination of all operations in primary and community care outside the acute hospitals, including the private providers, and to secure critical PPE material to them”. (12)*

*”The coordination of primary care needed to be tightened and SLSO gave an offer [to the regional emergency organisation] to do this, which resulted in a formal mandate to manage the whole operative coordination of the healthcare outside the regional acute hospitals”. (7)*

### Managing is to readjust and improve

#### Continuous adaptation

The managers stressed that the processes needed continuous adjustments and rearrangements. Activities were tightly followed-up to ensure maximum flexibility. The emergency management team members were located in the same room and had frequent meetings and took decisions rapidly when needed. Members expressed that they had to solve even ambiguous situations and be prepared to adjust if new information required changes. Some specialty services adopted digital tools originally developed for primary care to limit the risk of infection spread among their patients.

#### Information to support management

To provide up to date information for each function was an important task of the emergency management team. Information needed to be accurate and to make people feel safe and activate relevant responses. It was crucial to eliminate conflicting or outdated information on the intranet, the number one information channel.

#### Work processes

An important task for the emergency team members was to coordinate the operations of all emergency functions (listed earlier) and enable rapid responses. One example was the stock-piling and distribution of PPE and pharmaceuticals, early taken over by SLSO from the region.

#### Clear goal

The big picture was shared by everyone, thanks to active communication, and the clear goal to control the pandemic by relevant responses in a timely manner made all work in the same direction.

#### Large individual responsibility

The roles of the emergency management team members were assigned based on individual competence, personal networks and experience. Members were expected to take responsibility and to be creative and proactive to solve problems independently but within given limits.

#### Wide system responsibility

The emergency team members expressed that their organisation took a large responsibility for the whole system. The initiative to coordinate all primary and community care outside hospitals came from inside SLSO, not from the regional emergency management. SLSO realised the need for coordination in the field and acted. Some emergency management team members expressed that the regional level had complicated the implementation of the local emergency work, since decisions made were rapidly reversed with unclear roles and mandates as a consequence. SLSO had to make suggestions to the regional level to get things to happen.

*”We have been affected by decisions taken by the regional emergency organisation, but in the end we have had to make our own decisions to be able to do the right things, for example regarding test capacity”. (4)*

#### Teamwork and collaboration

The role of an emergency management team member was to support teamwork, and, in some cases, also to manage the operations in a clinic or service. Therefore, some emergency management team members were recruited externally because of their specific competence to take on such a responsibility. In normal situations the SLSO services are networking widely with other providers, which greatly facilitated the establishment of the coordinated “cluster organisation”. Documentation of all actions was done for formal reasons but was also seen as a source for learning and future evaluation.

**Fig. 3:**
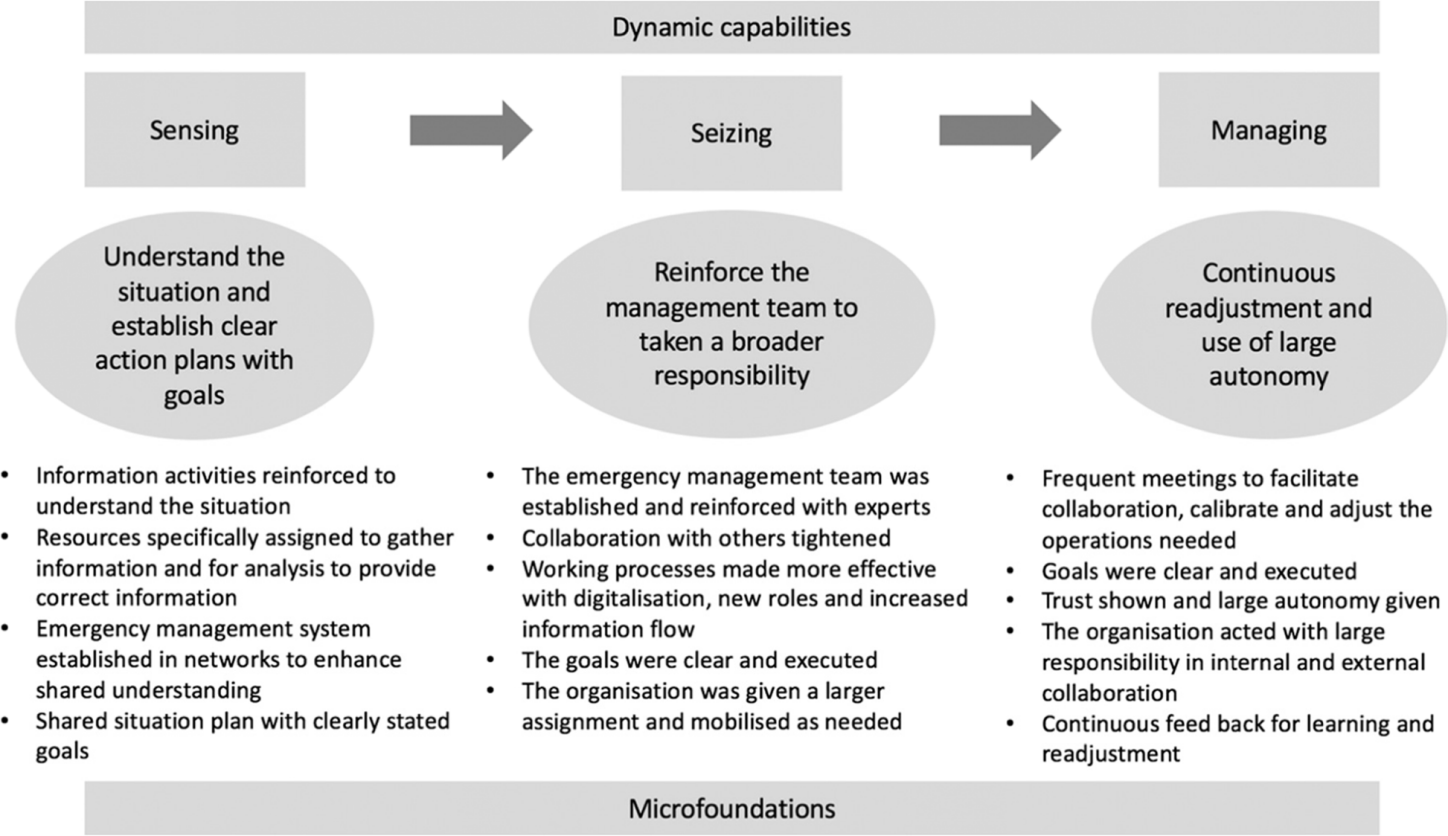
Overview of dynamic capabilities of emergency management

These results are summarised and organised according to the dynamic capability framework in Fig. 3 [15].

### Outcomes perceived by the emergency management team

#### Need for system effectiveness to meet future crisis

The emergency team members felt that good collaboration with external partners and stakeholder is important but it is also essential to preserve the improved internal coordinated way of working. The crisis has been a driving force for an improved collaboration between SLSO and the municipalities. The cooperation with private providers and the regional purchasing office has contributed to SLSO’s ability to flexibly and effectively navigate in the whole system. The focus should now be broadened from acute care to also include tailored plans for patient groups in priority order. The role of a future emergency organisation should be more succinctly defined.

#### Need for organisational effectiveness to meet future crisis

The SLSO emergency management showed its value and lessons learned should form plans for the future. The emergency management team was better equipped to lead crisis operations than the regional emergency organisation. The SLSO team was engaged and could make rapid changes with confidence, which contributed to a feeling of safety and control in the organisation. However, attention has to be paid to emergency management training and the recruitment of right persons, paying attention to both competence and personal characteristics. Improving organisational resilience is important in preparation for a future crisis. Personal assignments with plans for back-up and substitution as well as individual crisis support are important since working periods might be long and intense (as during this crisis).

#### Strategic organisational effectiveness after the first wave

Managers suggested that the organisation needs to continue to reinforce the internal collaboration between services. Some expressed that the contracts with the purchaser do not promote collaboration and should be adjusted accordingly. The emergency management team should be broader with more operative input from clinical directors and focus more on core healthcare development than on administrative issues. Some were worried that such an opportunity would be missed when the organisation is back to normal.

#### Operational effectiveness within the organisation

The managers expressed a feeling of confidence and safety. The trust for each other had been strengthened. The regional emergency model was basically functional but needs to be adjusted. For instance, meetings in the emergency management team were effective and could be held short due to their high frequency – a procedure that needs to be preserved. The precautions against infections and observing the risk that many team members and staff contract the disease should have been acknowledged at the start when the team was located in small venues.

#### Communication and information efforts need to be better calibrated

Communication and information have been perceived to function very well. However, the information sources were many and a lot of redundant information was a problem before better coordination was reached. Sometimes the information was perceived as too excessive and the big picture could have been presented more clearly to all employees to increase trust.

#### Operational effectiveness was achieved on system level

A number of strategic lessons on regional level to increase responsiveness were reported: to shorten the decision routes, to coordinate information from all sources, to improve the regional supply chain for PPE and pharmaceuticals and to clarify the cluster organisation in future emergency plans.

### The value of management decentralisation

#### Perceived large individual decision space and shared accountability

The emergency management team members’ mandate and accountability were ambiguous at start and some were not used to make decisions on their own and take personal responsibility. However, some members expressed that SLSO was used to delegated authority and trust-based management, which was reinforced and helped to manage this emergency. Trust has promoted problem solving in the cluster organisation. The emergency management team leader has shown a lot of trust to members, paid attention to all important questions and delegated the operative work to the appropriate emergency functions. There was no “blame game”, which was described as a key factor to encourage persons to come forward. One member said that no directives were given to the unit managers, but they were provided with support and shown trust which increased their sense of responsibility.

“Trust based steering is a factor that has enabled the cluster organisations to solve their challenges and create their own models to create commitment” (7)

#### Institutional capacity was strengthened by the collaboration across the region

The emergency team members expressed that when SLSO mobilised a proper emergency organisation the feeling of confidence and trust increased rapidly. Those responsible for communication gave appropriate information in a timely way. It was suggested to continue collaboration across clinic and service boarders to increase learning. The fact that emergencies were successfully met will mean that experience gained will be of use in possible future crises. Forms for he fruitful collaboration with other stakeholders, which needs to continue, will prepare the organisation well for the future. The “digital explosion” was identified as something that gives more leverage to the organisation and will be used to free time and resources. The media reporting was mentioned as an initial problem, because it redirected people’s attention from epidemiological or other scientific facts, to be paid attention to when planning new communication strategies. A shared view of problems is a must, promoted by a shared analysis of the situation based on facts, and is best performed in a cohesive way on the systems level.

*“The role of the SLSO emergency management team was unclear in relation to the regional emergency organisation and the other local emergency management teams at the hospitals”. (11)*

### Unit managers perceptions of changes on unit level

The 1,766 collated survey free text answers resulted in a total of 625 meaning units relevant to our aim, and were grouped into seven categories: Actions to achieve strategic effectiveness (247), Actions to achieve operational effectiveness (282), Descriptions of decision space (25), Descriptions of accountability (13), Institutional capabilities (28), Individual capabilities (3), and Other (27). Detailed information on these findings is available upon request.

Unit managers expressed that actions were taken that improved their units’ operational and strategic work. They fully utilised their decision space, well in congruence with their accountability, and were supported by the organisation to achieve these changes.

#### Unit managers mentioned measures that were taken to increase their units’ operational effectiveness

Initially, rapid changes were made to operations at unit level. Later but still during the first wave of the pandemic further changes stemmed from local or other contextual needs. Changes were also made to the units’ managerial routines. Frequent local unit emergency management team meetings were held, often daily, to make work processes more efficient. Examples are task shifting among staff, switch to online booking systems, and simpler administration for sick leaves. Measures were taken to ensure the physical health of the units’ workforce, such as limiting the number of physical visits, installing plexiglass shields, and increasing the use of personal protective equipment.

Units met patient needs, still maintaining safety, by increasing visits at a distance via video link and/or telephone. Video and telephone visits surged, substituting traditional physical visits. One manager expressed that their unit’s previous work using the organisation’s own digital platform had facilitated the transition to increased digital services. Furthermore, several experienced that video and telephone consultations led to fewer late cancellations, increased accessibility, improved continuity of care, time savings, and successful contacts with patients who were afraid of seeking help. Some mentioned that the crisis had led to a tipping point in staff attitudes to digital care with a greater willingness to continue using video- and telephone consultations. Concerns were expressed over the decline in physical visits because video or telephone visits were not adequately reimbursed. Managers also felt that those with low digital literacy would be at a disadvantage, increasing inequality.

*“We are not reimbursed for physician telephone visits even though these often times exceed physical visits in its content and time.. This leads to age discrimination. In order to receive adequate reimbursement for the time and effort we put in, we need to schedule older people for physical visits. Younger ones who can handle a smartphone are provided with video visits.”*

The unit managers expressed that they faced barriers to preserve beneficial changes after the crisis has passed. These challenges were policies related to the preference for physical visits, technological challenges, and central quality measurements that do not match the care provided. Several mentioned the risk of returning to old work habits once the crisis is over.

#### Measures were taken to create strategies to meet the demands of the pandemic – and beyond

Internal and external collaboration increased through geographic cluster formations to manage staffing and information sharing which was appreciated by many of SLSO’s unit managers and was mentioned as something they wished would continue after the pandemic. However, many had experienced barriers to continue the collaboration and feared that it would ultimately diminish due to too diverse agreements with the purchaser regarding reimbursement and areas of responsibility, as well as time pressure, silo thinking, and the new collaborative networks still being too weak.

To meet the demands stemming from the crisis, unit managers received and managed a large amount of information from many different sources in the healthcare system. They expressed that the information dispatched was generally appreciated but that the information flow was often intense and led to misunderstanding regarding, i.a., whose decision they were obliged to follow and what new routines for hygiene and personal protective equipment were issued. Some suggested that better integrated information from different sources, including the National Public Health Authority, could reduce misunderstandings.

#### “Patients get cranky and irritated when we don’t know what so tell them after the government has made different statements”.

Unit managers mentioned that staff expressed feelings of stress and anxiety related to the crisis, and that managing this became a large part of their daily job. Many told about overarching strategies created to manage staff’s anxiety and stress, such as educational efforts to minimise infection in the workplace, flexible work hours, working from home, and free parking for staff to minimise the risk of infection when traveling to work. Measures were also taken to maintain staff mental health, largely through transparent and frequent communication. Several mentioned daily meetings for sharing information and airing concerns, staff having high accessibility to unit managers through either an”open door policy” in the office or via telephone, and activities related to stress management, such as safe group sessions with a psychologist, separate meetings with staff members, encouraging breaks and rest in spare time, and safe group discussions regarding time to recover.

#### Unit managers’ decision space was utilised

Some managers mentioned the decision space they had utilised when making changes to the way their units delivered care due to the crisis. They either made decisions on their own or together with fellow managers and/or staff in the extended “cluster organisation”. The balance between central control and local decision space was described as follows:

#### “The decision to start an infectious node came from higher levels, but the processes and routines came from the staff”.

Three managers mentioned that some central instructions have been delivered too late after local solutions have already been developed which sometimes led to prolonged decision-making times or turning to non-standardised routines. Some expressed that they were affected by decisions made by actors elsewhere in the system and needed central support to preserve their decision space.

*“Due to late or contradictory directives regarding the management of patients and staff following a Covid infection, medically driven units have created their own routines, while other units wait for directives and are affected hard”*

#### Units’ degree of accountability increased

Several unit managers experienced an increased responsibility as to achieving the unit’s goals, such as providing care for patients in ways that were not necessarily reimbursed, or taking local initiatives, such as distributing a mental well-being survey to staff to ensure that their needs were met. However, several managers mentioned the need for central financial support to reach performance targets as well as the need for clarification regarding areas of responsibility when setting up new work structures.

*“Now the professionals get increased responsibilities and the opportunity to solve the problems that arise…… We get to use the knowledge and experiences we have, and even though the situation is new to us, it shows how high our competence and creativity is. We get to focus on what has always been our drive when working in healthcare, and we avoid micro-management, bureaucracy and financial focus, which we often experience otherwise. We get to find solutions here and now while being responsible for our whole unit. It strengthens our team spirit!”*

#### SLSO’s support system was mostly available when needed

Unit managers mentioned the support they had received from SLSO’s support system. Several of them expressed that they had positive experiences of support from central management. Examples cited were ordering PPE, additional transportation, support for treatment, help from human resources regarding rostering, and legal advice regarding staff rights. Three managers wrote that the support from central management was appreciated, and that the organisation had mobilised effectively, been proactive, and avoided catastrophic scenarios. However, one manager mentioned that it was often difficult to find the right contact person for central support due to rapid changes of staff. Two other mentioned that it was difficult to know who has the ownership and manages the decisions mentioned in information dispatched and that central management ought to clarify messages when needed.

## Discussion

The aim of management decentralisation within a healthcare organisation is to improve resource utilisation to better match to patients’ needs and adapt to local conditions. The focus is on *organisational performance and efficiency* as shown in empirical studies of service delivery organisations outside the hospital environment [4, 5]. In this study we have had the opportunity to explore if a decentralised service provider showed organisational *agility* when struck by unexpected turbulence caused by the outbreak of the Covid-19 pandemic,

Our analysis of emergency management team members’ perceptions in the interviews and the unit managers’ free text survey answers showed a highly dynamic organisation demonstrating both high operational effectiveness and capacity to strategically reposition rapidly. The decision space of managers as well as the organisation’s institutional capacity contributed to these changes, for which managers at central and local level were held accountable.

The emergency management team was rapidly mobilised and established an early analytic capacity. This enabled the organisation to understand how to structure the internal emergency management system in terms of procedures, operations and communication as well as to establish a network for collaboration with external actors. The fact that the emergency management team so rapidly was mobilised, that it understood the situation and took a large responsibility to mitigate spread and ensure care of patients in a safe way has been a reassuring proof of the organisations capacity and ability to readjust, despite the size of the organisation and its high number of services providing units. The organisation showed a high grade of dynamic effectiveness.

The emergency team members succeeded in creating a feeling of safety and clarity. The decision paths were shortened, the internal and external collaboration was strengthened, meetings were effective and the trust in each other was increased. The unit managers emphasised that a large degree of autonomy and decision space with delegated authority was given on a trust basis. The decentralised model made it possible to be focused with a high grade of operational effectiveness, but it also through feed-back and learning made it possible to reach new strategic positions, such as establishing the cluster organisation.

Abrahamsson and Brege [14] argue that high operational effectiveness is not only aimed to achieve a more rational resource use to meet the unexpected situations better but is also a way to drive strategic effectiveness in order to reach new positions to better respond to changes in the environment, which in turn reinforces the operational effectiveness. We make the claim that this dynamic was demonstrated in our case also.

In our interviews we have shown that the emergency management team activated dynamic capabilities, by rapidly identifying (sensing), structuring (seizing) and mobilising (managing) resources [15], resulting in high operational effectiveness. In addition, it used information from the weekly survey to the unit managers to make strategic decisions of importance for clinical operations. This modus operandi further increased dynamic effectiveness and made the organisation even more responsive. An example is the highly effective operative collaboration between private and public providers to better handle the separation of patients with suspected Covid-19 infection from other patients in order to hinder disease spread, which later led to a strategic decision to establish a permanent organisational body for coordination. A giant leap in digitalisation to handle the need for consultations in a pandemic environment is another example, which also has been reported by others [27, 28].

Trust is mentioned several times in the interviews of the emergency management team. In recent years the interest in the impact of trust in organisations has been more prominent in organisational research [29]. That research emphasisis that organisations characterised by a high grade of trust between employees, but also between employees and management, are more successful in different aspects [29]. Teamwork, commitment and strong values for the directions and the goals are elements identified as success factors [29]. These are all factors also mentioned by our interviewees.

A metaphor used by the political scientist Rothstein [30], *“the social trap”,* can be described as follows: “everyone” is a winner if “everyone” collaborates, but if you do not trust that the others, “everyone”, collaborate, it is meaningless to collaborate, since it requires that nearly “everyone” does so. It can be rational not to collaborate if you do not trust that “the others” also collaborate. The conclusion is that an effective collaboration can only be reached if the trust is mutual and that without this the “trap” closes, the organisation becomes ineffective even though “everyone” realises that it would be better to collaborate [30]. Our findings confirm not only the mutual trust between the members in the emergency management team, which they described as a success factor that even was strengthened through intense team-work, but it is also consistent with the unit managers’ perception of support from the emergency management team described as stable, timely, reassuring and clear on directions.

In emergency and disaster response management process-oriented approaches are used to a wide extent to ensure efficiency [31]. However, a drawback is that response processes prepared in advance usually are impeded by unexpected contexts, unique processes, temporal urgency or other surprising events ([31], p 967). Complexity perspectives have been introduced in disaster response management as a way to handle the dynamics of a disaster to enhance effectiveness and flexibility [32]. Coordination under unpredictable conditions is a challenge, and information is critical [33, 34]. Comfort ([35], p 194) define coordination as “aligning one’s actions with those of other relevant actors and organizations to achieve a shared goal.”

We define decentralisation as a management practice where service delivery managers receive and exercise delegated authority to achieve high service performance within specified limits to their authority and are held accountable for doing so.

This is based on the theory that the four most important factors explaining effective decentralisation are 1) delegated authority, clearly specified in relation to different sources, with limits to this authority specified, 2) the capacity of the manager to appropriately exercise this authority which is a function of their individual competence and the system´s capacity to provide the support they need, 3) effective accountability for performance, which means a) operating within limits such as standards, including those requiring coordination, and b) exercising authority appropriately to achieve high service performance and 4) a culture of norms that support using initiative to meet local needs and achieve high service performance [13].

As expected, referring to earlier studies on management decentralisation (4,5) we identified high operational effectiveness in the case organisation, also during the turbulent times of the pandemic. Thanks to their delegated authority with a vast decision space both emergency management team members and unit managers were able to meet needs with adequate actions. Emergency management team members were effective in in their designated functions which they were assigned due to their competence and experience. The unit managers expressed that they were able to use their delegated authority to make decisions fast to cope with situations that could not be handled higher up in the organisation.

The combined strategic and operational effectiveness thus emerged from the delegated authority and the responsibility shown by managers understanding their accountability when taking actions to respond to changing conditions and requirements. However, this process did also create a unique opportunity of learning in the organisation that strengthened not only the individual but also the institutional capacity of the organisation [13].

**Fig. 4:**
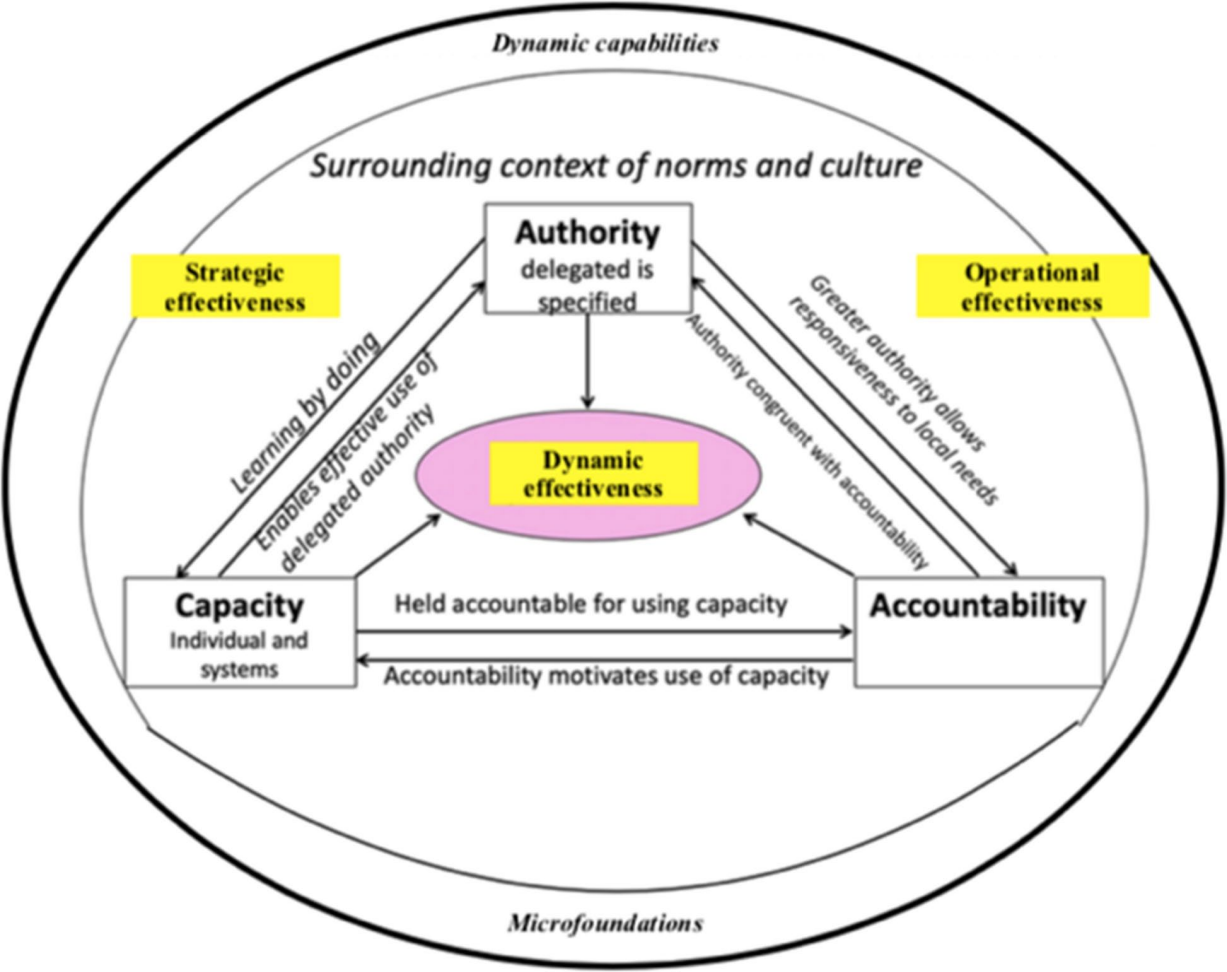
The Teece framework, Abrahamsson and Brege conceptual model and the revised Bossert model combined

In summary, the demonstrated strategic and operational effectiveness reinforced the overall dynamic effectiveness of the organisation as it mobilised dynamic capabilities as a platform for agile management enabled by the decentralised organisation defined by delegated authority, accountability and individual and organisational capacity. These interrelations are shown in Fig. 4.

Our findings showed the importance of building capacity on an institutional level for the emergency management of the whole organisation. Recent research has shown this to be necessary for a successful implementation of decentralisation and to ensure an equal standard of services provided [3, 36–39]. Lack of coordination, or that of the right type, is a threat to equality and may, in an emergency situation, lead to wrong priorities [40–43].

Improved performance is related to empowered and trained managers that are held accountable and encouraged to improve healthcare [44]. Decentralisation is not considered to be an end in itself, but a process to achieve the goals of efficiency and responsiveness. A problem is that benefits achieved in one environment cannot be taken for granted when transferred elsewhere without paying attention to crucial contextual factors [36].

Consequently, when building institutional capacity and enlarging decision space proper attention has to be paid to the context [45]. Responses to new decentralised structures might be both positive and negative, calling for readiness to make adjustments. “No man is an island.” Teamwork on all levels based on trust in a mutual relationship is shown both in previous research, as in our study to be of crucial importance.

## Conclusion

Unpredictable and turbulent events like the outbreak of the Covid-19 pandemic demand a high degree of agility from a healthcare organisation.. The ability to identify, use and manage resources as dynamic capabilities to launch needed actions is crucial. This is related to operational effectiveness, which in turn is dependent on the given decision space. A high degree of operational effectiveness will not only ensure proper responses to situational challenges but will also be a driver for strategic effectiveness to reach new strategic positions to even better cope with changing demands. The new strategic positions will strengthen the organisational capacity, which is a crucial component to ensure coordination in a decentralised model.

This study is, to our knowledge, the first of its kind where a decentralised management model in a service delivery organisation in healthcare is studied in relation to crisis management. Even though performed in one organisation, our findings, when analysed through lenses of dynamic effectiveness and dynamic capabilities, suggest that a decentralised model can be of importance to reach the organisational agility needed in a crisis.

## Supplementary Information

**Additional file 1. **

**Additional file 2. **

## Data Availability

The datasets used and/or analysed during the current study, data associated, and patient survey are available from the corresponding author on reasonable request.

## Abbreviations

HRM: Human Resources Management
PPE: Personal Protective Equipment
SLSO: Stockholms läns sjukvårdsområde (Stockholm Health Care Services
