# Supplementary material for: No man is an island: management of the emergency response to the SARS-CoV-2 (COVID-19) outbreak in a large public decentralised service delivery organisation
Source: BMC Health Serv Res. 2022 Mar 21;22:371. doi: 10.1186/s12913-022-07716-w (PMC8935606; doi:10.1186/s12913-022-07716-w)
Supplement: Supplementary file 1 — Additional file 1. [file 12913_2022_7716_MOESM1_ESM.docx]

Interview guide SLSO Local Emergency Management Team

Emergency response on Covid-19

Interview guide for semi-structured research interviews

Introduction

Thank you for taking time to help us with this research with the purpose to improve the emergency readiness when crisis situations like this happen. My name is___________

and I work within the research project “Evaluation of the emergency management organisation in SLSO”.

For the moment we interview persons that have been involved in the redesign of primary and community care as a response to the Covid-19 pandemic. The purpose with the project is to describe and evaluate the implementation of the changes in primary and community care as a response to the outbreak of Covid-19.

The aim is to follow the implementation process of an emergency management organisation druing the first weeks and months after the outbreak for learning as much as possible. The result will be presented to the organisation and in scientific articles.

The interview today will take 30 to 45 minutes and during this time I would like to ask questions about the changes that you have done as a response to the Covid-19 pandemic, what we can learn from each other and which of the changes should be permanented.

To facilitate the analysis process the interview will be recorded. Do you consent to this?

At the start of the interview I will ask you to give permission to this. Now I will start the recording after your given permission.

(Start recording)

My name is________________ Today it is (date and time) ___________________

and I interview (name)________________________________________________

I would like you to start to confirm that you have received full information about the project “Evalutation of the emergency management organisation in Stockholm Health Care Services”. Your participation is voluntary and you can withdraw at any time from the interview during or afterwards if you wish. All data is confidential and will be presented on group level. Your name will not appear in any document. Only the research group will have access to the data. You will get the possibility to read through the material that will be presented in our project.

Do you consent that this recorded interview will be used anynomised for research purposes?

□ Yes

□ No

Questions

You are invited to this interview because you have a leading role in the emergency management team (EMT) in SLSO.

1. What is your role in EMT.
2. How do you percieve your role as a leader in EMT?
   1. What do you think is expected from you in this role?
   2. How do you percieve your role and your responsibility/assignment in relation to the other working streams in EMT?
3. Can you describe the working processes of EMT and how it has developed over time?
   1. How do you think EMT has functioned (*Good or Bad)*?
   2. With the knowledge you have today – what would you like to preserve and bring to a new emergency management team?
   3. Now when we will finish the EMT work – what is the most important that we do today that you would like to continue doing after the crisis?
4. From your perspective, what factors do you think have facilitated the establishment and the execution in the EMT?
   1. In what ways have they been facilitating.
5. What factors do you think have been complicating or constraining to establish and execute in the EMT?
   1. In what ways have they been constraining?
6. How do you perceive the information on the Insidan [internal webb] and in ‘letters to the managers’?
   1. What information is most useful for you?
   2. What type of information do you miss?

Final questions

1. Given what we have talked about in the interview, is there anything you would like to add? Are there questions or topics we should bring to coming interviews?
2. Is there anyone more in the EMT or any clinical manager/managers you would like to recommend us to interview for better understanding?
   *Ask for contact information to these persons*

We would like to do a follow up interview this coming autumn and hope that we can contact you again. Thankyou for your time!
