# Supplementary material for: No man is an island: management of the emergency response to the SARS-CoV-2 (COVID-19) outbreak in a large public decentralised service delivery organisation
Source: BMC Health Serv Res. 2022 Mar 21;22:371. doi: 10.1186/s12913-022-07716-w (PMC8935606; doi:10.1186/s12913-022-07716-w)
Supplement: Supplementary file 2 — Additional file 2. [file 12913_2022_7716_MOESM2_ESM.docx]

Appendix 2

Unit manager survey free text questions regarding change work due to Covid-19 spring 2020

1) (a) What measures have you taken at your unit(s) in the past week due to the Covid-19 situation? (b) Possible instructions given regarding these measures? (Asked during period T1-13)

2) Are there any new instructions that complicate or prevent the implementation of important changes required by the Covid-19 situation? The instruction below complicates och prevents us from making the following changes: (Asked during period T1-13)

3) (a) Have there been considerably fewer requests for care from any specific patient group? Please, comment. (b) Do you believe these patients’ health has deteriorated due to the current situation? (Asked during period T9-13)

4) (a) Which type of patient are you most worried for? (b) What do these patients need most in order to prevent their health from deteriorating? (c) Do you have any ideas for what we, or others, can do to meet these needs during the upcoming weeks? (d) What would facilitate for you, or others, do do this? (Asked period T11-13)

5) (a) Do you take any measures to maintain the health of the patients who do not seek care? (b) Do you need any support/help to do this? If yes, what do you need help with? (Asked period T9-13)

6) What type of support do you most require from the SLSO emergency management team? (Asked during period T1-13)

7) (a) Has your unit changed its ways of working in a way that is important for utilising its current resources in a better way? If yes, please give examples of changed work processes that are important to keep. (b) What obstacles do you see for keeping these work processes? (Asked during period T8-13)

8) (a) Have you collaborated with other care divisions or professions during this period that you believe valuable to keep? If yes, please give examples of the other care divisions or professions with whom you have collaborated and would like to keep? (b) What obstacles do you see for keeping this collaboration from developing further? (Asked during period T8-13)

9) (a) Have you taken any measures to maintain your staff’s health? If yes, what measures have you taken? (b) How effective have these measures been? Please comment. (Asked during period T1-13)

10) Do you need any support from the SLSO emergency management team to maintain your staff’s health? If yes, what do toy need support with? (Asked during period T1-13)
